# Time use studies, time, temporality, and measuring care: Conceptual, methodological, and epistemological issues

**DOI:** 10.1177/0961463X231208981

**Published:** 2023-10-30

**Authors:** Andrea Doucet

**Affiliations:** Brock University, Department of Sociology and Women's & Gender Studies, St. Catharines, Ontario, Canada

**Keywords:** time use, care, relational time, care responsibilities, temporality

Across the globe, the COVID-19 pandemic has brought renewed attention to how people take on, manage, and divide their paid work and unpaid care work and responsibilities. It has also shone a spotlight on the enduring need for responsive and robust methodological and theoretical approaches to measuring and assessing unpaid care work. Perhaps the most trusted approaches for studying unpaid work are time use methods, with time use diaries being viewed as “the ‘gold standard’ for the measurement of routine activities,” including household work and unpaid care work (Altintas and Sullivan, 2016: 456). It is thus not surprising that time use methodologies have received increasing attention in the last few years (e.g., Charmes, 2015, 2019; Esquivel, 2011; UNSTATS, 2021) and especially since the first pandemic lockdowns in 2020 (e.g., Folbre, 2021; UNESCAP, 2021).

Although considerable methodological advancements have been made in time use methods over the past decade, assessments and critiques about the efficacy and applicability of time use studies for measuring the intersections between time and care have been growing. These critiques highlight, for example, the need to attend to the challenges of measuring overlapping categories and activities in everyday life; the relational and processual characteristics of time and care practices and concepts; the multiple meanings and enactments of time and temporalities in different contexts; the epistemological and ontological moorings of time use studies (Bryson, 2008); the gaps between theories of time and methodological approaches to time (e.g., Adam, 1989, 1995, 2006, 2018; Bryson, 2008; Cheng, 2017; Daly, 2002; Davies, 1994, 1996; Maher, 2009); and specific measurement challenges of studying practices, meanings, and spatial boundaries of care work, unpaid work, and community-based care work (e.g., Doucet, 2000, 2023; Doucet and Klostermann, 2023; Taylor, 2016). The papers in this special issue seek to expand time, temporalities, and time use studies discussions, debates, and critiques, especially those related to unpaid care work.

The contributions in this special issue heed the call to attend to the conceptual, theoretical, methodological, and/or epistemological complexities of assessing and measuring unpaid care work as well as to how care time and temporalities are experienced by diverse populations. It brings together work from Canada, Singapore, and the United States by several leading writers on care, paid and unpaid work, gender divisions of housework and care, time use studies, and time and temporality. The papers explore concepts and meanings of “time with” and “time for” children; what “in your care” means when measuring parental time; measurement dilemmas when studying care responsibilities; approaches to researching how people live and experience time through multiple conceptions of time; and temporalities and spatial and transnational dimensions of time.

Bookending this special issue are articles by Nancy Folbre and Andrea Doucet that address broad historical, methodological, and epistemological issues related to the measurement, categorization, classification, and conceptualizations of time and time use methods in studies on unpaid care work. Folbre's review article “Beyond the clock: Rethinking the meaning of unpaid childcare in the U.S” presents a unique conceptual history of childcare across more than a century of neoclassical economic theories, sociologies of work, 19th and 20th century studies in home economics, and varied histories of time use studies in the United States. Working with an expansive definition of childcare (that includes the work of supervision, socialization, management, and active face-to-face care), she explores how parental childcare has been particularly difficult for researchers to measure and assess and she lays out recent innovations in time use studies that grapple with these challenges. Folbre provides a “state-of-the art” overview of new methodological developments in international and comparative time use studies, points to connections between time use and qualitative research, and explores policy implications of time use studies. She also reflects on how her position on measuring housework and childcare has shifted since her earlier studies on gender divisions of household labor (e.g., Bittman et al., 2003). Among other recommendations, Folbre (this issue, p. 367-384) calls for greater “efforts to combine qualitative with quantitative methods” as these “could yield a richer picture of the meaning of ‘care’ as well as the meaning of “work” and she urges researchers to “look beyond the clock to carefully consider how time use categories are conceptualized” (p. 367-384).

Melissa Milkie and Dana Wray's article “Beyond mothers’ time in childcare: Worlds of care and connection in the early life course” is informed by an intersectional and life course approach and analyzes data (2014–2019) from the American Time Use Survey (ATUS) and the United Kingdom Time Use Survey (UKTUS). Moving beyond parental care, and particularly care provided by mothers, Milkie and Wray assess time diaries from children, teenagers, and mothers to highlight the community-based dimensions of time and to underscore the importance of focusing analyses on wider worlds of care beyond parental care. They advocate a “linked lives” approach that centers children's time spent in “socially connected” care and invite time use researchers to “rethink and measure other forms of time,” (p. 385-410) including the care work involved in building and maintaining social connections. Milkie and Wray introduce conceptual innovations to time use studies that highlight the co-presence of parents and others and how co-presence changes across the life course; they also widen and deepen specific time use measures such as children and teenagers’ “waking time alone and with others” (p. 385-410). As they argue: “By centering care time in the larger community of others from both mothers’ and children's perspectives, the analysis makes the case for an accounting of socially connected time as care using the rich and detailed measures available in time use surveys” (Milkie and Wray, this issue, p. 385-410)

The next two papers in this special issue are based on longitudinal qualitative research projects and explore the implications of their findings for rethinking theoretical, methodological, and epistemological conceptions of time in time use studies. Although these two papers and their informing projects do not work directly with time use methods, their critical reflections from diverse theoretical and epistemological positions offer valuable insight about shifting contextual and conceptual meanings of care, time, space, and temporalities.

“The critical temporalities of serial migration and family social reproduction in Southeast Asia” is written by Brenda Yeoh and co-authors Theodora Lam, Bittiandra Chand Somaiah, and Kristel Anne Fernandez Acedera. Based on a study from a larger mixed-methods project, this article draws on three phases of qualitative interviews conducted between 2009 and 2017 in rural communities in Indonesia and the Philippines with high levels of out-migration. It addresses the complexities of space, time, and temporalities for family migration within the current global context of rapidly rising temporary migration. It also explores “family times” and “times of migration” for transnational families from Indonesia and the Philippines, bringing attention to the limitations of the short-term temporal dimension of time use studies (i.e., questions about a typical day or days) and how different time measurement approaches and temporal and spatial frames (such as critical cross-generational temporal junctures across the life course) are needed for research with transnational migrant families. As Yeoh et al. (this issue, p. 411-433) write: “For many migrant women as well as men, migration decisions to leave, stay or return are intimately and temporally related to critical junctures and family life events, including birth and death, sickness and health, and the milestones marked by birthdays, the start of the school year, examination time, graduation, anniversaries, weddings and the arrival of grandchildren.”

Doucet's paper, “‘Time is not time is not time’: A feminist ecological approach to clock time, process time, and care responsibilities” is rooted in two interconnected research programs. The first is a qualitative and longitudinal research program (2000–2016) that included three Canadian studies focused on breadwinning mothers, fathers who identified as stay-at-home and/or primary or shared primary caregivers, and households where fathers took parental leave (mainly white, lower- and middle-income families, with some representation of diverse ethnicities and sexualities). The second program uses a feminist ecological and onto-epistemological approach that draws together relational and multiple ontologies and ethico-politico dimensions of knowledges and knowledge making. Focusing on how one case study couple (Lilly and Billy) described their care-work lives at two points over a decade, Doucet highlights acute conceptual and measurement challenges in studying care responsibilities. She argues that care responsibilities, which unfold as “process time” and “past-present-future time,” are relational and non-linear forms of time that can be narrated through qualitative research studies but cannot be measured in fixed units of clock time. Like Folbre's article in this issue, she advocates moving beyond the dominance of clock time to consider the diverse ways that people live and experience time and unpaid care work. She also highlights multiple relationalities and interdependencies within and between different kinds of time and calls for the recognition “that particular concepts, methods, epistemologies, ontologies—and more broadly, social imaginaries of knowledge making—will bring forth different kinds of time and that there can be ethico-political reasons for emphasizing one or more versions of time in relation to particular problematics” (Doucet, this issue, p. 434-460).

All four of the articles in this special issue ask us to rethink how we study and measure care work, unpaid work, and care processes with and through concepts, methods, and measures of time.
